# Acyl-CoA: cholesterol acyltransferases-2 gene polymorphism is associated with increased susceptibility to coronary artery disease in Uygur population in Xinjiang, China

**DOI:** 10.1042/BSR20182129

**Published:** 2019-02-26

**Authors:** Yong-Tao Wang, Buamina Maitusong, Yi-Tong Ma, Zhen-Yan Fu, Yi-Ning Yang, Xiang Ma, Xiao-Mei Li, Fen Liu, Bang-Dang Chen

**Affiliations:** 1 Department of Cardiology, First Affiliated Hospital of Xinjiang Medical University, Urumqi 830054, P.R. China; 2 Xinjiang Key Laboratory of Cardiovascular Disease Research, Urumqi 830054, P.R. China; 3 Unit of Cardiovascular Epidemiology, Institute of Environmental Medicine, Karolinska Institutet, Nobels väg 13, Box 210, 17177 Stockholm, Sweden

**Keywords:** ACAT-2 gene, coronary artery disease, polymorphism, susceptibility

## Abstract

Background: Acyl-CoA: cholesterol acyltransferases (ACAT) is the only enzyme that catalyzes the synthesis of cholesterol esters (CE) from free cholesterol and long-chain fatty acyl-CoA and plays a critical role in cellular cholesterol homeostasis. In the present study, our primary objective was to explore whether the single-nucleotide polymorphisms (SNPs) in ACAT-2 gene were associated with coronary artery disease (CAD) in Uygur subjects, in Xinjiang, China.

Methods: We designed a case–control study including 516 CAD patients and 318 age- and sex-matched control subjects. Using the improved multiplex ligation detection reaction (iMLDR) method, we genotyped two SNPs (rs28765985 and rs7308390) of ACAT-2 gene in all subjects.

Results: We found that the genotypes, the dominant model (CC + CT vs TT) and over-dominant model (CT vs CC + TT) of rs28765985 were significantly different between CAD patients and the controls (*P*=0.027, *P*=0.012 and *P*=0.035, respectively). The rs28765985 C allele was associated with a significantly elevated CAD risk [CC/CT vs TT: odds ratio (OR) = 1.48, 95% confidence interval (CI) = 1.02–2.16, *P*=0.04] after adjustment for confounders. The TC and LDL-C levels were significantly higher in rs28765985 CC/CT genotypes than that in TT genotypes (*P*<0.05).

Conclusions: Rs28765985 of ACAT-2 gene are associated with CAD in Uygur subjects. Subjects with CC/CT genotype or C allele of rs28765985 were associated with an increased risk of CAD.

## Introduction

Coronary artery disease (CAD) is a common cardiovascular disease and has been the leading cause of death in the world wide [1]. CAD is recognized as a complex disease and is caused by multiple risk factors, including smoking, chronic inflammation, immune diseases, hypertension, diabetes and hyperlipidemia [2–6]. In the past decades, with the development of genome technology, genome wide association study (GWAS) and the next-generation sequencing (NGS) were more and more used in etiology research and a number of genetic susceptibility genes associated with the risk of CAD have been discovered [7–9].

Acyl-CoA: cholesterol acyltransferases (ACATs), known as sterol O-acyltransferases (SOATs), are microsomal proteins responsible for catalyzing the synthesis of intracellular cholesterol ester (CE) from free cholesterol and long-chain fatty acyl-CoA and thus play an important role in cellular cholesterol homeostasis [10]. There exist two subtypes of ACAT, ACAT-1 is ubiquitously expressed in a variety of tissues while ACAT-2 is expressed in a species-specific manner and exclusively expressed in enterocyte of the intestine and hepatocyte of the liver [10]. One of the important roles of ACAT-2 is to provide CEs for lipoprotein assemblies, including chylomicrons and very low-density lipoproteins (VLDL) [11].

Previous studies have found that deletion of ACAT-2 may delay the development of atherosclerosis in animal models. Researchers found that both global restricted and tissue-restricted ACAT-2 gene deletions mice had significantly lower levels of intestinal cholesterol absorption and reduced percentages of hole sterol palmitate and cholesterol oleate in LDL CE. Further histological experiment also showed that deletion of ACAT-2 gene in LDLr/ mice significantly delayed the development of atherosclerosis [12–14]. All these suggested that ACAT-2 play an important role in cholesterol metabolism and genetic variations in the ACAT-2 gene may be associated with the individual’s susceptibility to CAD.

Genetic factors may affect CAD via distinct mechanisms and an individual with a certain gene polymorphism may be more susceptible to CAD [15,16]. Therefore, gene polymorphism might be not only an independent risk factor of CAD but also biomarkers for the diagnosis, treatment and prognosis of CAD in precision medicine. However, little is known about the association between ACAT-2 polymorphisms and CAD. The present case–control study aimed to explore the association of ACAT-2 gene polymorphisms with CAD in a Uygur population.

## Methods

### Ethical approval of the study protocol

The present study was approved by the Ethics Committee of the First Affiliated Hospital of Xinjiang Medical University (Xinjiang, China). It was conducted according to the standards of the Declaration of Helsinki. All of the patients provided written informed consent and explicitly provided permission for DNA analyses, as well as for the collection of relevant clinical data.

### Subjects

The analyses were carried out in a case–control study design. A total of 834 subjects (516 diagnosed CAD cases and 318 healthy controls) of Uygur were recruited from the First Affiliated Hospital of Xinjiang Medical University between August 2010 and October 2016. CAD was defined as presence of at least one significant coronary artery stenosis of >50% luminal diameter based on the coronary angiography. In the present study, we only collected patients with stable angina as CAD group, and patients with acute coronary syndrome were excluded. Patients with concomitant valvar heart disease, congenital heart disease and/or no ischemic cardiomyopathy were also excluded. All control subjects were selected from volunteers who had angiographically normal coronary arteries and had no history of CAD [17]. Coronary angiography in the control individuals was performed for the evaluation of chest pain. Individuals were excluded from the present study if they had: a history of CAD; electrocardiographic signs of CAD; regional wall motion abnormalities; relevant valvar abnormalities in echocardiograms and/or carotid atherosclerosis. Hypertension was defined as a systolic blood pressure ≥ 140 mmHg and/or a diastolic blood pressure ≥ 90 mmHg at least on two distinct occasions. Diabetes mellitus was defined as two fasting plasma glucose level ≥ 7.0 mmol/l. The following information was collected: age, gender, hypertension, diabetes, total cholesterol (TC), triglyceride (TG), high-density lipoprotein cholesterol (HDL-C) and low-density lipoprotein cholesterol (LDL-C).

### Genotyping

Fasting blood samples drawn via venipuncture in the catheter room were taken from all participants before cardiac catheterization. The blood samples were drawn into a 5-ml ethylene diamine tetraacetic acid (EDTA) tube and centrifuged at 4000 × ***g*** for 5 min to separate the plasma content. Genomic DNA was extracted from the peripheral leukocytes using the standard phenol–chloroform method. The DNA samples were stored at −80°C until use. For use, the DNA was diluted to a concentration of 50 ng/μl. Using Haploview 4.2 software and International HapMap Project website phase I &II database (http:// www.hapmap.org), we obtained four tag SNPs of ACAT-2: SNP1 (rs28765985) and SNP2 (rs7308390), by using minor allele frequency (MAF) ≥0.05 and linkage disequilibrium patterns with *r*^2^ ≥ 0.8 as a cutoff. The SNP genotyping was performed using an improved multiplex ligation detection reaction (iMLDR) technique (Genesky Biotechnologies Inc., Shanghai, China). The primers for the polymerase chain reaction (PCR) and the probes for the LDR were listed in Table 1. Genotyping was performed in a blinded fashion without knowledge of the patients’ clinical data, and a total of 10% of the genotyped samples were duplicated to monitor genotyping quality.

**Table 1:** The primer sequences for each SNP

SNPs	Primers or probes	Sequences
rs28765985	Upstream primer	CCCCAGAGTAGCTTCTGTTGTAATAGC
	Downstream primer	GACAGGATTTCTCCATATTGGTCAGG
	RC	TCTCTCGGGTCAATTCGTCCTTTCAGGCTGGTCTCAAACTCACG
	RP	ACCTCAGGTGATCTGCCCGTTTTTTTTTTTT
	RT	TGTTCGTGGGCCGGATTAGTTCAGGCTGGTCTCAAACTCACA
rs7308390	Upstream primer	
	Downstream primer	
	RC	TTCCGCGTTCGGACTGATATCACATCTGCCTCAGGGCAAGTTG
	RP	TCATTGAGAAGCATTTGAGARTGGCTTTTTTTTTTTTTTTTT
	RT	TACGGTTATTCGGGCTCCTGTCACATCTGCCTCAGGGCAAGCTA

### Statistical analysis

The data analysis was performed using SPSS version 17.0 for Windows (SPSS Inc., Chicago, IL, U.S.A.). The Hardy–Weinberg equilibrium was assessed via Chi-square analysis. The measurement data are shown as the means ± SD, and the differences between the CAD subjects and the control subjects were assessed using an independent-sample *t*-test. Differences in the enumeration data, such as the frequencies of smoking, drinking, hypertension and ACAT-2 genotypes between the CAD patients and the control subjects, were analyzed using the Chi-square test. Additionally, logistic regression analyses with effect ratios (odds ratio [OR] and 95% CI) were used to assess the contribution of the major risk factors. The dominant model is defined as heterozygote+homozygote variant vs. homozygote wild; the recessive mode is defined as homozygote variant vs. heterozygote+homozygote wild; the overdominant model is defined as heterozygote vs. homozygote wild and homozygote variant vs. homozygote wild respectively; the codominant model is defined as heterozygote vs. homozygote variant +homozygote wild. A *P* value < 0.05 was considered to be statistically significant

## Result

### Characteristics of subjects

The baseline characteristics of 516 CAD patients and 318 control subjects were shown in Table 2. The mean age, BMI, TG levels and the prevalence of female, smoking and drinking were similar between CAD patients and the controls (all *P*>0.05). The CAD patients and controls differed significantly with regards to hypertension (*P*=0.002), diabetes mellitus (DM) (*P*=0.009), TC (*P*=0.002), HDL-C (*P*=0.014), LDL-C (*P*=0.001) and glucose (*P*=0.001).

**Table 2:** Clinical and metabolic characteristics of subjects

Characteristics	Control (*n*=318)	CAD (*n*=516)	*χ*^2^ or *t*	*P* value
Age, mean (SD)	55.98 ± 10.02	56.85 ± 10.05	1.219	0.223
Sex, female (%)	108(34.0)	155(30.0)	1.403	0.236
Hypertension, n (%)	123(38.7)	256(49.6)	9.486	0.002
Diabetes, n (%)	6(1.9)	29(5.6)	6.821	0.009
Smoking, n (%)	85(26.7)	166(32.2)	2.769	0.096
Drinking, n (%)	55(17.3)	104(20.2)	1.043	0.307
BMI, mean (SD)	26.71 ± 3.99	26.76 ± 3.59	0.22	0.826
TG, mean (SD)	1.82 ± 1.23	1.95 ± 1.08	1.546	0.123
TC, mean (SD)	4.09 ± 1.30	4.39 ± 1.32	3.119	0.002
HDL-C, mean (SD)	0.96 ± 0.27	0.90 ± 0.33	2.453	0.014
LDL-C, mean (SD)	2.70 ± 0.81	2.94 ± 1.07	3.49	0.001

Continuous variables are expressed as mean ± SD. Categori cal variables are expressed as percentages.

The *P* value of the continuous variables was calculated by the independent samples *t* test. The *P* value of the categorical variables was calculated by *χ*^2^ test.

Abbreviations: BMI, body mass index; HDL-C, high-density lipoprotein-cholesterol; LDL-C low-density lipoprotein-cholesterol; TC, total cholesterol; TG, triglyceride.

### Distributions of genotype and allele in CAD patients and controls

Table 3 shows the distribution of genotypes and alleles for the two SNPs (rs28765985 and rs7308390) of the ACAT-2 gene. The genotype distributions of the two SNPs were in accordance with the Hardy–Weinberg equilibrium in controls (all *P*>0.05). For rs28765985, the distribution of the genotypes, the dominant model (CC + CT vs TT), the overdominant model (CT vs CC + TT) showed significant differences between CAD patients and the controls (*P*=0.027, *P*=0.012 and *P*=0.035, respectively). Whereas there were no significant differences between CAD patients and controls in the distribution of rs7308390 genotypes, dominant model, recessive model and overdominant model (*P*=0.776, *P*=0.488, *P*=0.73 and *P*=0.545, respectively).

**Table 3:** Distribution of SNPs of ACAT-2 gene in CAD and controls

Genotype	Model		Case (*n*, %)	Control (*n*, %)	*P*	Crude OR (95% CI)	*P*
rs28765985	Codominant	TT	396 (76.7)	267 (84.0)	0.027	1	0.031
		CT	110 (21.3)	49 (15.4)		1.514 (1.045–2.193)	0.028
		CC	10 (1.9)	2 (0.6)		3.371 (0.733–15.508)	0.119
	Dominant	TT	396 (76.7)	267 (84.0)	0.012	1	0.013
		CT+CC	120 (23.3)	51 (16.0)		1.586 (1.104–2.280)	
	Recessive	TT+CT	506 (98.1)	316 (99.4)	0.123	1	0.143
		CC	10 (1.9)	2 (0.6)		3.123 (0.680–14.344)	
	Overdominant	CC+TT	406 (78.7)	269 (84.6)	0.035	1	0.036
		CT	110 (21.3)	49 (15.4)		1.487 (1.027–2.154)	
rs7308390	Codominant	CC	427 (82.8)	269 (84.6)	0.776	1	0.776
		CT	81 (15.7)	45 (14.2)		1.134 (0.764–1.683)	0.533
		TT	8 (1.6)	4 (1.3)		1.260 (0.376–4.225)	0.708
	Dominant	CC	427 (82.8)	269 (84.6)	0.488	1	0.488
		CT+TT	89 (17.2)	49 (15.4)		1.144 (0.782–1.674)	
	Recessive	CT+CC	508 (98.4)	314 (98.7)	0.73	1	0.731
		TT	8 (1.6)	4 (1.3)		1.236 (0.369–4.139)	
	Overdominant	CC+TT	435 (84.3)	273 (85.8)	0.545	1	0.545
		CT	81 (15.7)	45 (14.2)		1.130 (0.761–1.676)	

Tables 4 and 5 show the multivariable logistic regression analyses of the major confounding factors for CAD. Following the multivariate adjustments for the confounders, such as age, sex, BMI, TG, TC, HDL-C, LDL-C and prevalence of hypertension, DM, smoking and drinking, rs28765985 is still an independent risk factor for CAD [CC/CT vs. TT: odds ratio (OR) = 1.48, 95% confidence interval (CI) = 1.02–2.16, *P*=0.04]. Whereas after adjustment for the confounders, rs7308390 is still not the independent risk factor for CAD (*P*=0.383).

**Table 4:** Results of logistic analysis (rs28765985)

	OR	95% CI	*P* value
rs28765985 (CT+CC vs. TT)	1.483	1.018–2.161	0.04
Gender	1.189	0.855–1.654	0.304
Age	1.007	0.992–1.022	0.367
BMI	0.985	0.947–1.025	0.461
Hypertension	1.515	1.122–2.046	0.007
TG	1.063	0.932–1.213	0.364
TC	1.146	1.007–1.303	0.038
HDL-C	0.485	0.294–0.800	0.005
LDL-C	1.284	1.068–1.542	0.008
Diabetes	2.739	1.105–6.794	0.03
Smoking	1.294	0.869–1.925	0.204

**Table 5:** Results of logistic analysis (7308390)

	OR	95% CI	*P* value
rs7308390 (CT+TT vs. CC)	1.193	0.803–1.774	0.383
Gender	1.181	0.849–1.642	0.322
Age	1.006	0.991–1.021	0.417
BMI	0.982	0.944–1.021	0.363
Hypertension	1.513	1.121–2.041	0.007
TG	1.066	0.934–1.217	0.342
TC	1.155	1.015–1.315	0.029
HDL-C	0.471	0.287–0.775	0.003
LDL-C	1.31	1.090–1.574	0.004
Diabetes	2.699	1.089–6.690	0.032
Smoking	1.273	0.856–1.891	0.233

### Stratified analysis between ACAT-2 gene polymorphisms and CAD risk

Gender, hypertension, smoking and drinking are the most common risk factors of CAD. To further analyze the interaction between CAD and environment, we performed stratification analyses in terms of gender, hypertension, smoking and drinking status to evaluate how these variables modified the association between the SNPs (rs28765985 and rs7308390) and CAD risk (Table 6). For smoker, the dominant model (CC/CT vs. TT) of rs28765985 remains significantly associated with CAD (OR = 2.41, 95% CI = 1.06–5.48, *P*=0.036). For drinker, the dominant model (CC/CT vs. TT) of rs28765985 remains significantly associated with CAD (OR = 3.17, 95% CI = 1.06–9.45, *P*=0.036).

**Table 6:** Stratified analysis between ACAT gene polymorphisms and CAD risk

	Case/control	rs28765985 (Genetype)	Adjusted OR (95% CI)	*P* value	rs7308390 (Genetype)	Adjusted OR (95% CI)	*P* value
Gender							
Male	361/210	CT+CC/TT	1.524 (0.963–2.413)	0.072	CT+TT/CC	1.583 (0.987–2.540)	0.057
Female	155/108	CT+CC/TT	1.800 (0.878–3.689)	0.108	CT+TT/CC	0.566 (0.243–1.318)	0.187
Hypertension							
No	260/195	CT+CC/TT	1.538 (0.939–2.520)	0.088	CT+TT/CC	1.360 (0.798–2.317)	0.59
Yes	256/123	CT+CC/TT	1.546 (0.844–2.829)	0.158	CT+TT/CC	1.078 (0.588–1.979)	0.807
Smoking							
No	350/233	CT+CC/TT	1.342 (0.863–2.086)	0.192	CT+TT/CC	0.914 (0.555–1.506)	0.725
Yes	166/85	CT+CC/TT	2.406 (1.057–5.479)	0.036	CT+TT/CC	2.027 (0.997–4.121)	0.051
Drinking							
No	412/263	CT+CC/TT	1.364 (0.904–2.057)	0.139	CT+TT/CC	1.012 (0.645–1.588)	0.96
Yes	104/55	CT+CC/TT	3.171 (1.064–9.445)	0.038	CT+TT/CC	1.897 (0.793–4.539)	0.15

### Genotypes and serum lipid levels

**Figure 1:**
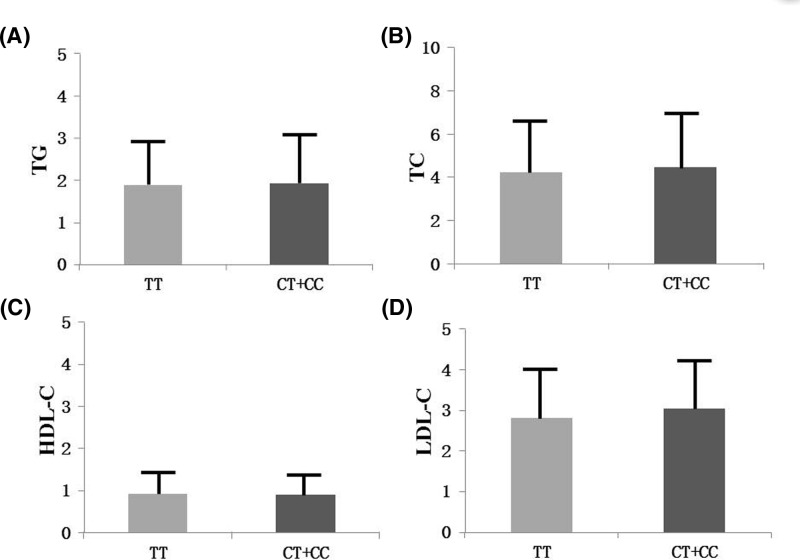
Association between rs28765985 and lipid parameters (**A**) There exists no significant difference of TG between rs28765985 CC/CT genotypes and TT genotypes (*P*>0.05). (**B**) The TC levels were significantly higher in rs28765985 CC/CT genotypes than that in TT genotypes (*P*<0.05). (**C**) There exists no significant difference of HDL-C between rs28765985 CC/CT genotypes and TT genotypes (*P*>0.05). (**D**) The LDL-C levels were significantly higher in rs28765985 CC/CT genotypes than that in TT genotypes (*P*<0.05).

As shown in Figure 1, the TC and LDL-C levels were significantly higher in rs28765985 CC/CT genotypes than that in TT genotypes (*P*=0.028 and *P*=0.003 respectively).

## Discussion

In the present study, we investigated associations between two SNPs in the ACAT-2 gene and CAD risk in a Chinese Uygur population. Our results show that rs28765985 is significantly associated with CAD susceptibility. Our findings also demonstrated significant associations between rs28765985 of ACAT-2 gene and TC and LDL-C concentration.

ACAT is the only enzyme that catalyzes the synthesis of CE from free cholesterol and long-chain fatty acyl-CoA in cells and plays an important role in the absorption, transport and storage of lipids such as cholesterol and fatty acids [10]. ACAT has always been thought to be drug targets for therapeutic intervention of several diseases including atherosclerosis, cancer, Alzheimer and gallbladder disease [18–21]. There exist two subtypes of ACAT in mammalian, ACAT-1 and ACAT-2. These two subtypes are quite different with regard to gene chromosomal locations, tissue-specific expression and substrate specificity. ACAT-2 is specifically expressed in hepatocytes and enterocytes, where CE can be incorporated into very-low-density lipoprotein (VLDL), as well as cytoplasmic lipid droplets. Research have demonstrated that increased cholesteryl ester secretion occurs in livers of animals fed with cholesterol and is closely related to the occurrence and extent of coronary artery atherosclerosis. All these suggested that ACAT2-derived cholesteryl esters may promote arterial cholesterol accumulation during coronary artery atherosclerosis.

The development of atherosclerosis in ACAT-2 deficiency was first studied by Willner et al. [13]. They investigated the contribution of ACAT2-derived cholesterol esters in ACAT-2^–/–^ /ApoE^–/–^ mice model and ACAT-2^+/+^/ApoE^–/–^ (control) mice model. Although ACAT-2^–/–^ /ApoE^–/–^ mice and ACAT-2^+/+^/ApoE^–/–^ mice had similar elevated plasma apoB and total plasma lipids, the main components of the lipid are different. The primarily lipid cores of the apoB-containing lipoproteins were triglycerides, but not CE, in ACAT-2^–/–^ /ApoE^–/–^ mice. Further, ACAT-2^–/–^ /ApoE^–/–^ mice also had lower level of aortic atherosclerosis compared with controls. These dates suggested that the amount of CE exists in the core of lipoproteins is apparently more important in atherosclerosis. Whereas ACAT-2 deficiency in mice may restrict synthesis of CE and reduce the accumulation of CE in the plasma lipoproteins and atherosclerosis development in ACAT-2^–/–^/ApoE^–/–^ mice. Lee et al. [12] got the similar conclusion in LDLr^–/–^/ mice. They found that ACAT-2^−/−^ deficiency had a significant decrease of plasma CEs. ACAT-2^−/−^ deficiency mice lead to not only a 78% reduce of aortic surface area as lesion, but also 88% reduce of CE deposited in atherosclerotic plaques.

Previous studies have also demonstrated an atherogenic potential of ACAT2-derived CE in humans. Ma et al. [22] performed a study on the association of plasma phospholipid and cholesterol ester fatty acid composition to carotid artery intima-media thickness in 2872 participants and found that the carotid intima-media thickness was significantly associated with ACAT-2-derived CE in lipoproteins. Furthermore, Warensjö et al. [23] performed a community-based prospective study with a maximum follow-up of 33 years and found that ACAT2-derived CE in lipoproteins was significantly associated with CVD mortality. To identify the association of plasma CE levels derived from the ACAT-2 and acute coronary syndrome, Miller et al. [24] performed a single-center prospective cohort study in America. They found that ACAT2-derived CE in lipoproteins have strong potential value in the prediction of acute coronary syndrome.

The association between ACAT-2 gene polymorphisms and CAD and serum lipid levels was poor. He et al. [25] performed a study in 809 CAD patients and 1304 controls from three distinct Singaporean ethnic groups (1228 Chinese, 367 Malays and 518 Indians). They found that the 734C>T variant in ACAT-2 gene were significantly associated with plasma concentrations of apoA-1, apoB and lipoprotein (a) in Indians and with apoA-1 in Malays and the 734T allele frequency was significantly lower in CAD than that in controls in Chinese. The 41A>G variant in ACAT-2 gene is associated with total cholesterol in Indians. Chen et al. [26] performed a study to analyzed relationship between plasma HDL-C levels and putatively functional SNPs in 42 genes in a Caucasian population. They found that rs2272296 in ACAT-2 gene were independent genetic determinants of plasma HDL-C levels. From the above example, we have considered that the association between ACAT-2 gene and CAD and lipid levels was different on account of polymorphisms of the ACAT-2 gene and ethnic differences.

In the present study, we genotyped polymorphisms of rs28765985 and rs7308390 SNPs in the ACAT-2 gene and found that rs28765985 was associated with CAD and lipid levels. The rs28765985 CC/CT genotype has a higher frequency in CAD patients than that in controls. After adjustments for several confounders, this association remained exist, indicating that the rs28765985 CC/CT were independent risk factors for CAD and the risk of CAD was increased in the subjects with the C allele in rs28765985. Furthermore, we found that C allele (CC/CT) carriers in rs28765985 have higher levels of TC and LDL-C when compared with C allele non-carriers. These results might provide convincing evidence for assuming subjects who carry C allele in rs28765985 may have increased susceptibility to CAD than that who carry T allele.

Despite the promising findings in the present study, several limitations should be mentioned. First of all, we only drawed conclusions based on the present observational association study, we failed to get a cause-and-effect relationship between risk factors and CAD. Second, the sample size of present study is relatively moderate. The association between rs28765985 and CAD should be confirmed by studies with larger sample size. Third, the present study lacked functional validation. Additional studies need to be undertaken to clarify the underlying molecular mechanism that associates the ACAT-2 gene polymorphisms with CAD.

## Conclusion

The present study revealed that rs28765985 of ACAT-2 gene is associated with CAD in Uygur subjects. Subjects with CC/CT genotype or C allele of rs28765985 were associated with an increased risk of CAD. The CC/CT genotypes of rs28765985 were also associated with increased serum TC and LDL-C levels.

## Availability to data and materials

All data generated or analyzed during this study are included in this published article.

## Abbreviations

ACAT: Acyl-CoA: cholesterol acyltransferases
CAD: coronary artery disease
CE: cholesterol ester
GWAS: genome wide association study
HDL-C: high-density lipoprotein-cholesterol
LDL-C: low-density lipoprotein-cholesterol
SNP: single-nucleotide polymorphism
TC: total cholesterol
TG: triglyceride
VLDL: very low-density lipoprotein
